# Addressing the barriers to implementing model based designs in dose escalation studies

**DOI:** 10.1186/1745-6215-16-S2-P235

**Published:** 2015-11-16

**Authors:** Victoria Cornelius, Simon Bond

**Affiliations:** 1 King's College London, London, UK; 2 Cambridge University Hospitals NHS Foundation Trust, Cambridge, UK

## 

Dose escalation studies are undertaken to determine the recommended dose for later phase trials, often the maximum tolerated dose that can be administered beyond which the rate of toxicity events becomes too high. The advantages of model-based designs over rule based approaches (such as the 3+3 design) have been established for many years. While further innovations in design are being developed by the statistical community, a large number of trial investigators remain using the 3+3 design.

A one-day open meeting was held by NIHR Statistics Group in December 2014 to discuss the issues involved and gain consensus on a strategy to promote model-based methods. Two working groups have subsequently been developed. The first group are exploring the barriers to implementation and developing motivational examples targeted to the clinical community. The second group are providing pragmatic practical guidelines to the tools, choices and assumptions that go into planning and running a mode-based design study.

This poster will summarise the advantages of using a model-based approach, list the key perceived barriers for trial investigators to implement model-based methods and outline the work plan to address these barriers.

